# Feeling in Control: The Role of Cardiac Timing in the Sense of Agency

**DOI:** 10.1007/s42761-020-00013-x

**Published:** 2020-08-28

**Authors:** Aleksandra M. Herman, Manos Tsakiris

**Affiliations:** 1 Lab of Action and Body, School of Psychology, Royal Holloway University of London, Egham, Surrey TW20 0EX UK; 2 The Warburg Institute, University of London, London, UK

**Keywords:** Sense of agency, Intentional binding, Cardiac arousal, Interoception, Decision-making

## Abstract

The sense of agency describes the experience of controlling one’s body to cause desired effects in the world. We explored whether this is influenced by interoceptive processes. Specifically, we investigated whether the sense of agency changes depending on where, in the cardiac cycle (systole or diastole), the action was executed and where the outcome of the action occurred. In two experiments, participants completed decision-making task to win/lose money. Explicit (ratings of control) and implicit (temporal judgement) measures of agency were differentially affected by cardiovascular state. Implicit agency scores were affected by the cardiac phase at the point of action execution. Explicit ratings of control were affected by the type of (free vs. instructed) and by outcome valence (win vs. lose). The time of the action was uniformly distributed across the cardiac cycle. These results show interoceptive impact on agency, but that cardiac cycle may affect explicit and implicit agency differently.

**Electronic supplementary material:**

The online version of this article (10.1007/s42761-020-00013-x) contains supplementary material, which is available to authorized users.

## Introduction

The sense of agency (SoA) describes not only the experience of having a body but also the experience of controlling one’s body to cause desired effects in the environment (Haggard & Tsakiris, 2009). Synofzik, Vosgerau, & Newen (2008) suggested that SoA can be described at two distinct levels. While the high-level SoA (i.e., ‘judgement of control’) is conceptualized explicitly through one’s own interpretation of one’s agency, the low-level SoA (i.e., ‘feeling of control’) is experienced non-conceptually and pre-reflectively (Synofzik et al., 2008). In the experimental setting, the SoA can be measured explicitly by asking individuals whether they felt they caused an action or how much in control they felt (Haggard & Tsakiris, 2009) or, alternatively, by implicitly looking at distortions in time perception associated with agency—an effect known as *intentional binding* (Haggard, Clark, & Kalogeras, 2002). Intentional binding refers to the temporal attraction between an action (e.g., pressing a light switch) and its effects (i.e., light), so that if an action is followed by an outcome, the two events are perceived temporally closer together than in the control condition when the action and the outcome occur independently.

A recent theoretical model has proposed that interoception, defined as the ability to sense the internal state of the body (Craig, 2003), may influence both self-awareness and agency, with the sense of agency depending on the integration of internal bodily cues with exteroceptive sensory signals (Seth, Suzuki, & Critchley, 2012; Seth & Tsakiris, 2018). Furthermore, the mainteinance of homeostasis occurs through the adaptive change of the organism’s internal environment to meet perceived (and anticipated, i.e., allostatic) demands (Gu & FitzGerald, 2014; Pezzulo, Rigoli, & Friston, 2015; Sterling & Eyer, 1988). Importantly, interoception is thought to be a key element of allostasis (Barrett, Quigley, & Hamilton, 2016; Gu & FitzGerald, 2014). Only when we are able to detect our current bodily needs, we can adjust our behaviour accordingly (Gu & FitzGerald, 2014). Therefore, our actions and decisions are influenced by interoceptive processes and may also, themselves, have important interoceptive consequences (Quattrocki & Friston, 2014). Indeed, recent evidence provides a link between interoceptive and motor mechanisms, whereby accurate anticipation of the homeostatic feeling states that are associated with outcome valence facilitates motor execution and outcome evaluation (Marshall et al., 2019). Therefore, interoceptive processing is considered crucial for action generation, control, and self-attribution of action (Marshall, Gentsch, & Schütz-Bosbach, 2018). However, despite those theoretical links between interoception and agency, to our knowledge, the role of interoceptive signalling in the SoA has not yet been examined.

**Fig. 1:**
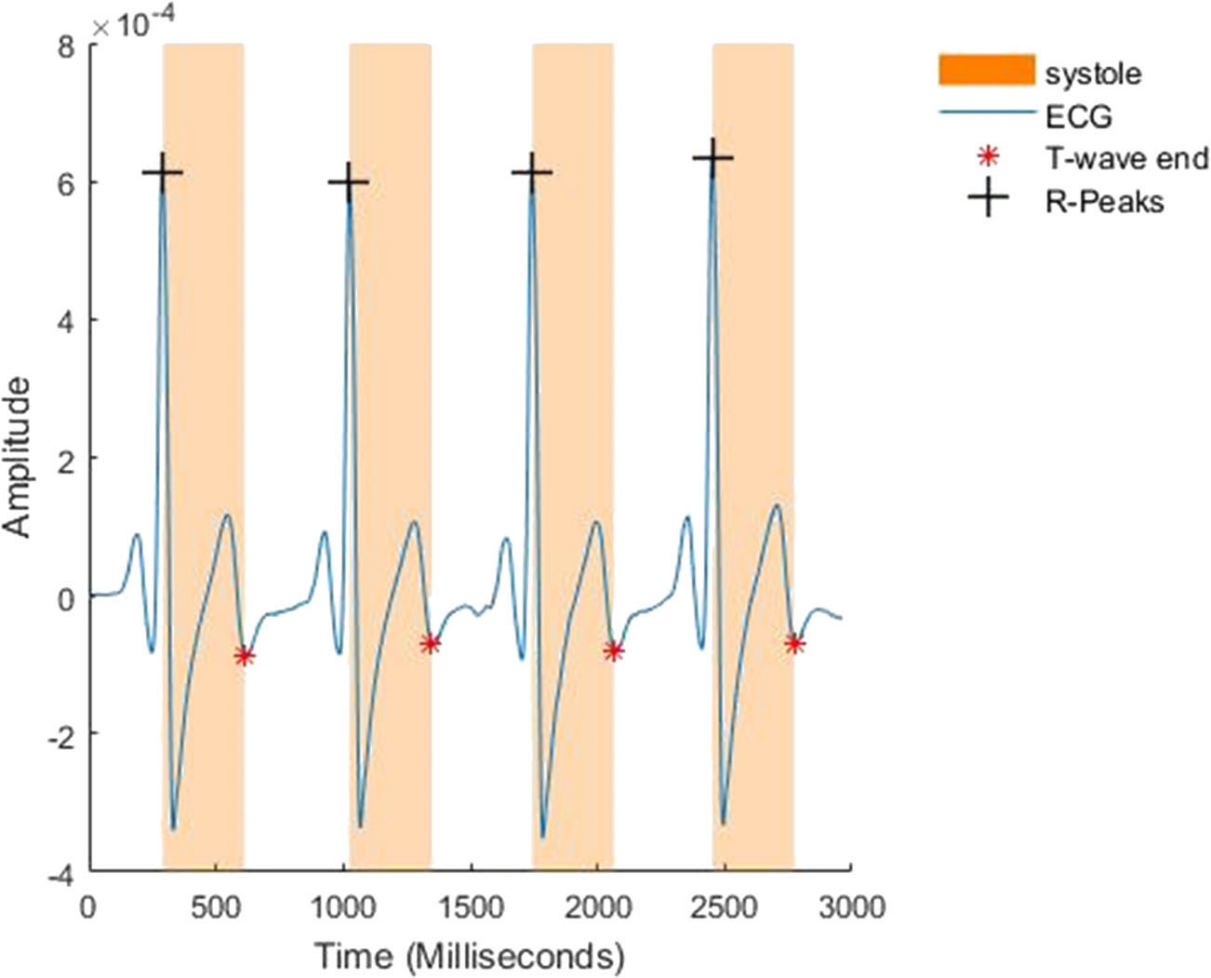
Sample ECG recording depicting four full cardiac cycles (from one R peak to another). Cardiac systole (in orange), the phase of increased cardiac arousal, starts at the R peak and last until the end of T-wave

There is a growing body of evidence suggesting that subtle bodily cues affect our perception of the world, our actions and decisions. Due to its regularity and frequency, cardiac afferent signalling is the most commonly studied bodily cue. The brain receives signals concerning the state of cardiovascular arousal, i.e., the timing and strength of each cardiac contraction, which encodes heart rate and blood pressure (e.g., stronger, faster heartbeats) (Critchley & Harrison, 2013; Garfinkel & Critchley, 2016). Arterial baroreceptors (pressure sensors), in the great vessels that leave the heart, show increased activity when the heart contracts to eject blood (at systole; Fig. 1). These neural signals carry information to the brain about the strength and timing of each heartbeat. Baroreceptors are quieter between heartbeats (at diastole). During states of elevated cardiovascular arousal, heart rate and blood pressure rise simultaneously, leading to an increased proportion of time spent in ventricular systole, because increases in heart rate occur mainly at the expense of the length of diastole. As a result of these changes, arterial baroreceptors are activated more strongly and for a longer period. In states of cardiovascular arousal (including emotional stress), the baroreflex is suppressed, allowing heart rate and blood pressure to rise together. While experimentally increasing heart rate induces prioritisation of fear processing across different measures (Pezzulo et al., 2018), similar results have also been observed by simply synchronising the presentation of fearful stimuli to cardiac systole (Garfinkel et al., 2014; Garfinkel & Critchley, 2016; Watson et al., 2019).

The synchronised presentation of brief stimuli at systole or diastole in experiments allows us to examine the impact of phasic baroreceptor activity (which convey information about the state of cardiovascular arousal) on central processing of stimuli, while avoiding the confounding effects that general psychophysiological arousal may have on perception and behaviour (Critchley & Garfinkel, 2015, 2017, 2018; Quadt, Critchley, & Garfinkel, 2018).

Notably, perceptual awareness of interoceptive processes and signals is not necessary for the visceral afferent signalling effects to occur. Therefore, cardiac timing experiments are illustrations of preconscious interoceptive impact. One can compare responses to brief stimuli presented around systole, when the baroreceptors are more active, to responses to stimuli presented at diastole when the baroreceptors are more quiescent. A brief stimulus presented at systole is processed concurrently with aortic/carotid baroreceptors signalling, while this is not the case for a stimulus presented at diastole (Lacey & Lacey, 1970; Rau & Elbert, 2001). Differences in processing that stimulus can then be attributed to the increased versus dampened cardiac interoceptive signalling.

Historically, baroreceptor stimulation was generally considered to be inhibitory (Lacey & Lacey, 1970; Lacey & Lacey, 1978). In some domains, systolic cardiac signals indeed show inhibitory effects on perceptual and cognitive processing. For example, systolic afferent signals suppress startle responses, attenuate memory encoding for words, and dampen subjective pain and somatosensory perception (Al et al., 2020; Garfinkel et al., 2013; Motyka et al., 2019; Schulz et al., 2016; Wilkinson, McIntyre, & Edwards, 2013). However, in other domains, these baroreceptor signals also show facilitatory effects. For example, the detection of rapidly presented visual stimuli, particularly conveying fear, is enhanced during cardiac systole compared to cardiac diastole (Garfinkel et al., 2014; Park, Correia, Ducorps, & Tallon-Baudry, 2014; Pramme, Larra, Schächinger, & Frings, 2016). Perception of disgust can also be enhanced to a smaller extent by systolic signalling (Gray et al., 2012; cf. Garfinkel et al., 2014). Moreover, race-driven misidentification of weapons is increased during systole, which is associated with enhanced negative racial stereotypes (Azevedo, Garfinkel, Critchley, & Tsakiris, 2017). In addition, the cardiac cycle appears to influence our actions. Pre-potent response inhibition in the stop signal task is enhanced if the onset of the stop cue is presented during cardiac systole rather than diastole (Rae et al., 2018). Early experiments employing simple reaction time paradigms also suggested that responses to stimuli which occurred during cardiac systole were slower compared to those which occurred at diastole (Birren, Cardon, & Phillips, 1963; Jennings & Wood, 1977; Lacey & Lacey, 1970). Later studies, however, showed this may be the case only in certain circumstances and depends, for example, on the response laterality, stimuli presentation location, the length of the heart period (Weisz & Adam, 1996). Additionally, more recent studies failed to replicate cardiac effects on response times (Rae et al., 2020, 2018).

Notably, as much as cardiac signalling can affect our actions, our actions, in turn, affect the heart: cardiac acceleration and deceleration are observed during action preparation and execution, respectively (Börger & van der Meere, 2000). Cardiac deceleration also occurs during the inhibition of movements (Jennings, Van der Molen, Brock, & Somsen, 1992; Van Der Veen, Van Der Molen, & Jennings, 2000; Van der Veen, Van der Molen, & Jennings, 2001).

Overall, cardiac timing experiments suggest that systolic signalling may have inhibitory or facilitatory effects on different aspects of perception and behaviour. We, therefore, focus here on whether interoceptive signalling affects the SoA over one’s choices and actions, an area of research that remains under-investigated.

### Aims

SoA is considered to depend on the integration of interoceptive cues with exteroceptive sensory signals (Seth et al., 2012; Seth & Tsakiris, 2018). Therefore, experimentally elucidating the role of such bodily cues is important for an understanding of how basic homeostatic processes impact on the sense of control and responsibility we take for our own choices and actions. We were interested to determine whether the SoA in decision-making is modulated by the phases of the cardiac cycle (cardiac systole and diastole) and whether the timing of action-making in relation to the cardiac cycle is also related to the SoA. To establish this, we developed a simple decision-making task, in which choice type (freely made or instructed choice), outcome type (win or lose) and the timing of outcome delivery (systole and diastole) were manipulated (experiment 1). In the subsequent experiment (experiment 2), while participants made (free) decisions to win or lose money, we investigated the role of the cardiac phase and the potential interaction of this with the timing of the delivery of the action outcome (systole and diastole). In line with past research (Barlas, Hockley, & Obhi, 2018; Kulakova, Khalighinejad, & Haggard, 2017; Moreton, Callan, & Hughes, 2017; Takahata et al., 2012; Tanaka & Kawabata, 2019; Yoshie & Haggard, 2017), we expected elevated agency in the free vs. instructed conditions and also following wins rather than losses, especially for the explicit agency measure. From the past evidence suggesting that cardiac systole is associated with enhanced perception of threat-related events (Azevedo et al., 2017; Garfinkel et al., 2014; Watson et al., 2019) and inhibition of motor responses (Rae et al., 2018), we hypothesised that systolic signalling would lead to diminished SoA judgements.

## Experiment 1

### Methods

In experiment 1, we examined the role of choice type, outcome valence and the timing of outcome delivery on both implicit and explicit measures of agency. We developed a decision-making task in which on each trial participants’ choices were either made freely or by following instructions, resulting in meaningful outcomes, in terms of monetary rewards or losses. Moreover, we manipulated the onset of the delivery of the outcome, whereby on half of the trials participants learnt about the outcomes when the heart was contracting (systole), eliciting baroreceptor afferent firing, compared to when the heart fills between beats (diastole) and when baroreceptors are thus quiescent.

### Participants

Forty-six participants (8 males; age 18–31, 20.57 ± 2.58 years old) were recruited from the Royal Holloway University subject pool. Inclusion criteria comprised: age between 18 and 35 years old, normal or corrected-to-normal vision and hearing, and no current treatment for any mental or neurological disorders. Participants in our sample were not taking any medication (except for birth control), and we did not observe any abnormalities in their ECG signal. The sample size was determined based on prior power calculations (*f*(*U*) set at 0.25, 1-*β* = 0.9, G*power 3.1). Participants gave written informed consent before taking part in the study and were reimbursed £10 per hour for their time, plus an additional £2 as winnings in the task (see below). All procedures were approved by the local Ethics Committee and were conducted following The Code of Ethics of the World Medical Association.

### Tasks

The tasks were programmed and delivered using Cogent 2000 Toolbox (Wellcome Dept., London, UK) in MATLAB (v2016a; Mathworks Inc.).

#### Cardiac Time Estimation Task

On each trial, participants heard a tone (100 ms duration) followed after a short delay by a sound (indicative of winning or losing, as in the decision-making task, see below). The second sound was presented either on cardiac systole (200 ms following the R peak) or cardiac diastole (500 ms following the R peak). Timing of stimuli presentation was based on previous research (Azevedo et al., 2017; Garfinkel et al., 2014; Gray, Rylander, Harrison, Gunnar Wallin, & Critchley, 2009), to maximise the chance of presentation during appropriate phases of the cardiac cycle. To ensure that, on average, the delays between systole and diastole trials were equal, on systole trials an initial delay was set to 300 ms, after which the R peak was detected. Participants had to estimate how much time, in milliseconds, had passed between the tone and the sound. Participants were informed that the delay between the sounds could be anything between 0 and 2500 ms, but no training on the task was provided, as we were mainly interested in general biases in time estimation rather than accuracy. The outcome variable was a *T*_score_ calculated from the formula: *T*_score_ = (ReportedTime – RealTime)/RealTime. *T*_scores_ were used in the analysis as they allow direct comparison of estimates of different durations. Negative values indicate underestimation, while positive values reflect overestimation of time.

Participants completed 40 trials in total: 20 ‘systole’ trials, 20 ‘diastole’ trials. On half of the trials, the tone was followed by the winning sound and on the other half by the losing sound.

#### Cardiac Risky Decision-Making Task

**Fig. 2:**
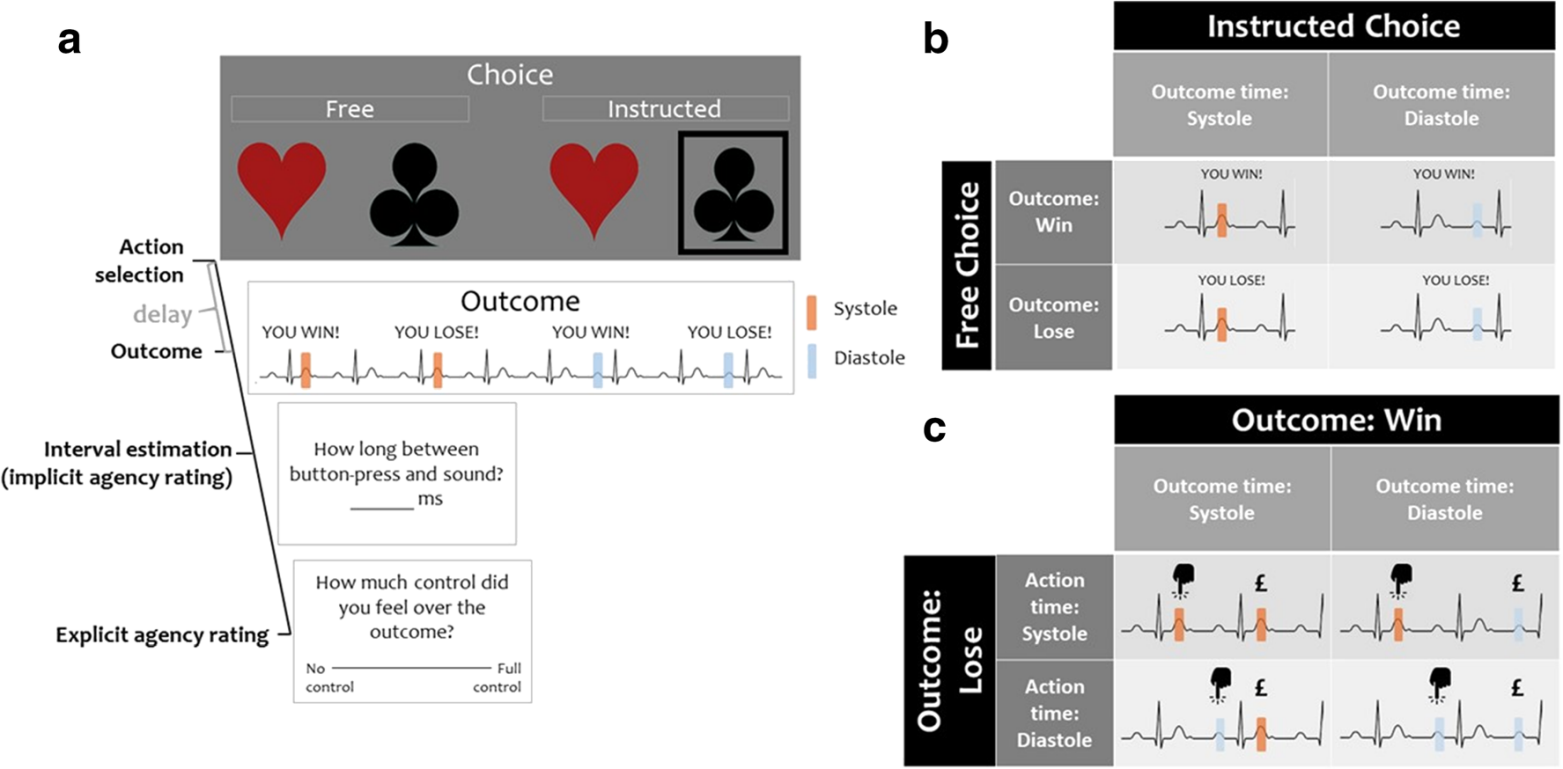
The task and design. **a** The cardiac risky decision-making task as used in experiment 1. On each trial, participants were choosing between two card suits presented on the screen. The choice was either free or instructed. Following a delay, the auditory outcome, indicating a monetary gain or a loss, was presented, either or cardiac systole or diastole. Next, participants judged in milliseconds, how much time passed between their response and the outcome presentation. Finally, they provided explicit control ratings. The task consisted of 160 trials. Simultaneous ECG was recorded to trigger the stimuli presentation in both experiments and to analyse behaviour relative to cardiac cycle in experiment 2. **b** Experiment 1 followed a 2 (choice: free vs. instructed) × 2 (outcome valence: win vs. lose) × 2 (outcome time: systole vs. diastole) design. **c** In experiment 2, only the free condition was used. The timing of each action was matched against the ECG recording to determine at which point within the cardiac cycle each action was made

The decision-making task was based on a task described by Kulakova et al. (2017). On each trial, participants were presented with a pair of card suits on a homogenous grey background (Fig. 2a). There were 12 possible pairs (i.e., four suits: clubs, hearts, diamonds and spades; in two locations: left or right). There were two conditions to the task: free and instructed. In the free condition, participants could select whichever symbol they thought was the winning one, using the respective arrow key. Participants were told that a correct choice would win them 20p, while incorrect choice would lose them 18p. In the instructed condition, one of the symbols was surrounded by a black frame. Participants were instructed to always choose the symbol in the black frame, to have 50% chance of winning. On these instructed trials, if the wrong symbol was selected, information was presented: ‘Incorrect symbol was selected. You lose 18p’. As incorrect responses suggest decreased attention, incorrect instructed trials were removed from the analysis.

The outcome of the decision was presented, after a delay, at cardiac systole (200 ms following the R peak) or cardiac diastole (500 ms following the R peak). To ensure that, on average, the delays between systole and diastole trials were equal, on systole trials an initial delay was set to 300 ms, after which the R peak was detected. The outcome of the decision was indicated with a sound (a winning or losing sound respectively) lasting 100 ms. Following the presentation of the outcome, participants had to estimate, in milliseconds, the time elapsed between the key press they made to indicate their choice and the outcome (sound) presentation. Participants were informed that the delay between the sounds could range between 0 and 2500 ms, but no training on the task was provided, as we were mainly interested in biases in individual responses rather than accuracy. Additionally, participants rated how much control they felt over the outcome, on a visual analogue scale (VAS) ranging from 0 (no control) to 100 (full control).

There were two outcome variables, reflecting implicit (time estimation) and explicit (VAS ratings) agency level. The implicit agency was reflected in a *T*_score_ (as described above) calculated for each condition. The higher the *T*_score_ the lower the sense of agency, and vice versa.

The presentation of different options on the screen was intended to induce an impression that some sort of rule to the task existed that could be applied to maximise the winnings, and thus to create the illusion of control (Stefan & David, 2013). In reality, the outcomes (win/lose) were presented in a (different) shuffled order for each participant, with each person experiencing 50% winning and 50% losing trials for each condition. At the end of the task, participants were paid their winnings and debriefed.

There were 160 trials in total, presented in two blocks of 80 trials, with a self-paced break to rest in between. The design (Fig. 2b) consisted of three fixed factors: choice (free vs. instructed) × outcome (win vs. lose) × cardiac cycle (systole vs. diastole). The task took approximately 35 min to complete.

### ECG Recording

Three disposable ECG electrodes were placed in a modified lead I chest configuration: two electrodes were positioned underneath the left and right collarbone and another on the participant’s lower back on the left side, within the ribcage frame. The ECG signal was recorded using a Powerlab 8/35 box (Bio Amp 132) and LabChart 8 software (https://www.adinstruments.com). The sampling rate was 1 kHz and a hardware band-pass filter between 0.3 and 1000 Hz was applied, as well as a 50 Hz notch filter to reduce electrical noise. During the tasks, heartbeats were detected online with the LabChart’s fast response output function, a hardware-based function that identifies the R-wave, with minimal delays (~ 1 ms), each time the ECG amplitude exceeds an individually tailored threshold.

The ECG recording was visually inspected for artefacts.

### Procedures

Participants first completed cTET as a baseline measure of time estimation. To familiarise participants with the task procedures, and ensure compliance with the instructions, six practice trials of the cardiac risky decision-making task followed. Then, participants completed the full version of the task.

### Data Analysis

#### cTET

The delay between the tones and the onset of sounds at systole and diastole were compared with paired samples *t* test. *T*_scores_ were analysed with 2 (sound valence: winning vs. losing) × 2 (sound time: systole vs. diastole) repeated measures ANOVA. The analysis was performed in JASP (JASP Team, 2020).

#### Cardiac Risky Decision-Making Task

VAS ratings and *T*_scores_, as dependent variables, were analysed with 2 (outcome: win vs. lose) × 2 (outcome time: systole vs. diastole) × 2 (choice type: free vs. instructed) repeated measures ANOVAs, followed up by post hoc paired samples *t* tests, as appropriate, performed in JASP (JASP Team, 2020).

**Table 1:** The results of the 2 × 2 × 2 repeated measures ANOVA comparing VAS ratings on the risky decision-making task. Significant p values (< .0.05) are depicted in italics

	Sum of squares	df	Mean square	*F*	*p*	*η*^**2**^
Choice	32,071.83	1	32,071.83	34.37	*< .001*	0.43
Residual	41,990.51	45	933.12			
Outcome time	22.05	1	22.05	1.53	.223	0.03
Residual	650.32	45	14.45			
Outcome valence	5942.49	1	5942.49	35.42	*< .001*	0.44
Residual	7549.23	45	167.76			
Choice × outcome time	89.83	1	89.83	5.16	*.028*	0.10
Residual	783.22	45	17.41			
Choice × outcome valence	0.42	1	0.42	0.02	.904	0.00
Residual	1283.18	45	28.52			
Outcome time × outcome valence	0.43	1	0.43	0.03	.858	0.00
Residual	595.63	45	13.24			
choice × outcome time × outcome valence	0.01	1	0.01	0.00	.988	0.00
Residual	943.35	45	20.96			

Type III sum of squares

A two-sided alpha level of 0.05 was used in all main statistical analyses. Bonferroni correction for multiple comparisons was applied for post hoc tests.

For completeness, we also computed inter-subject correlations between implicit and explicit agency measures for each condition. We have reported this analysis in the Supplementary Materials.

## Results

### cTET

There were no significant differences in delay between tones and sounds presented on systole vs. diastole (*t*(45) = 1.08, *p* = .288, *d* = .16; systole delay: *M* = 836.20 ms, SD = 70.41, diastole delay: *M* = 825.10 ms, SD = 68.13), indicating that any further differences in time estimation must arise from biases in time perception between the conditions.

There were no significant main effects: (cardiac cycle: *F*(1, 45) = 0.002, *p* = .962, *η*^2^ < 0.001; sound valence: *F*(1, 45) = 2.56, *p* = .117, *η*^*2*^ = .054)); nor an interaction (*F*(1, 45) = 2.22, *p* = .144, *η*^2^ = 0.05) on *T*_scores_, indicating that at baseline there were no significant differences in time estimation as a result of the sound timing (cardiac cycle phase at the sound onset) or the sound valence.

### Cardiac risky decision-making task

#### Explicit sense of agency

**Fig. 3:**
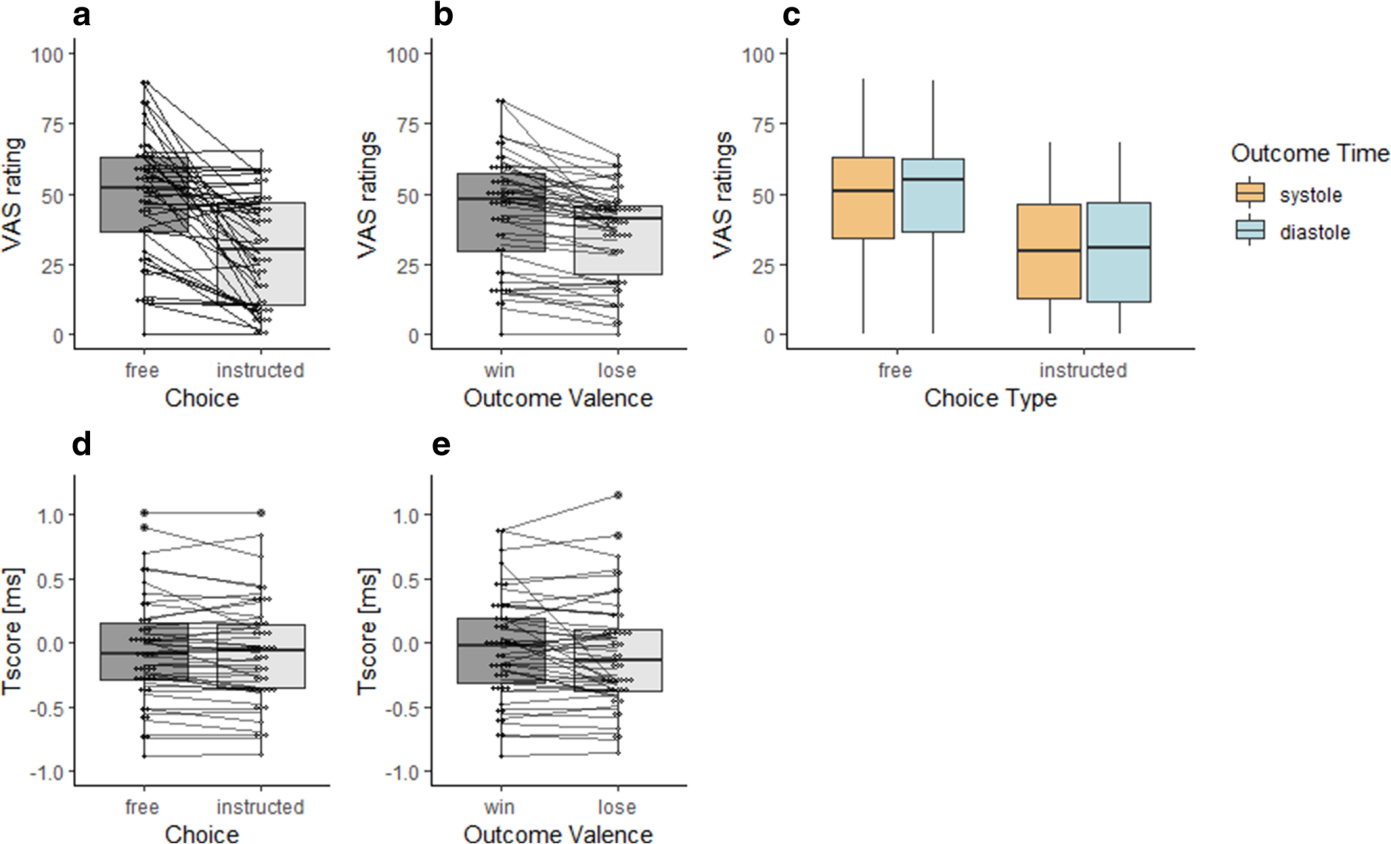
Results from experiment 1. The main effect on VAS control ratings in the decision-making task of choice type (**a**) and outcome type (**b**), as well as choice type x cardiac cycle interaction (**c**). Higher VAS score indicate higher sense of agency. The main effect of choice type (**d**) and outcome type (**e**) on *T*_scores_ (implicit agency measure). Higher *T*_scores_ indicate lower sense of agency

Cronbach‘s α (Cronbach, 1951) for VAS control ratings equalled 0.94 with a 95% CI of (0.92, 0.97), suggesting that the explicit agency measure has satisfactory internal consistency. There were significant main effects of choice, as well as of outcome valence on VAS ratings (Table 1, Fig. 3a and b), suggesting that individuals felt more in control in the free vs. instructed condition and also when they were winning vs losing money. In addition, there was a significant choice type × cardiac cycle interaction, indicating that participants reported equal agency when the outcomes were presented at systole vs. diastole in the instructed condition, but they reported higher agency ratings when the outcomes were presented at diastole vs. systole in the free-choice condition (Fig. 3c). Post hoc paired samples *t* tests revealed that VAS ratings were higher, in the free condition, when the outcome was presented at diastole rather than systole (*t*(45) = 3.06, *p*_uncorr_ = .004, *d* = 0.45), while there were no significant differences in VAS ratings in the instructed condition (*t*(45) = 0.56, *p* = .580, *d* = 0.08).

**Table 2:** The results of the 2 × 2 × 2 repeated measures ANOVA comparing *T*_scores_ on the risky decision-making task

	Sum of squares	df	Mean square	*F*	*p*	*η*^2^
Choice	0.08	1	0.08	3.84	.056	0.08
Residual	0.99	45	0.02			
Outcome time	0.00	1	0.00	0.08	.776	0.00
Residual	0.66	45	0.02			
Outcome valence	0.28	1	0.28	3.36	.073	0.07
Residual	3.77	45	0.08			
Choice × outcome time	0.02	1	0.02	1.00	.324	0.02
Residual	0.66	45	0.02			
Choice × outcome valence	0.01	1	0.01	1.40	.243	0.03
Residual	0.35	45	0.01			
Outcome time × outcome valence	0.01	1	0.01	0.63	.431	0.01
Residual	0.62	45	0.01			
Choice × outcome time × outcome valence	0.01	1	0.01	1.53	.222	0.03
Residual	0.36	45	0.01			

Type III sum of squares

#### Implicit Sense of Agency

Cronbach‘s *α* (Cronbach, 1951) for *T*_scores_ equalled 0.98 with a 95% CI of (0.9, 0.99), suggesting that the implicit agency measure has satisfactory internal consistency. There were no significant main effects or interactions in *T*_scores_ (Table 2). However, there was a trend for the main effect of choice type and outcome, suggesting that participants tended to judge the delays between the key press and the outcome presentation as longer in the free choice than instructed conditions (Fig. 3d). Moreover, participants tended to judge the delays between the key press and the outcome presentation as longer in the win vs. lose condition (Fig. 3e). Overall, these results suggest that participants’ implicit SoA was relatively lower in the free-choice vs. instructed-choice condition, as well as when they were winning vs. losing. Following Kulakova et al. (2017), we analysed option selection reaction times as potential confounding factors.

### Reaction Times

Decision reaction times (RTs) were defined as the interval between trial onset and key press. Paired samples *t* test (*t*(45) = 7.77, *p* < .001, Cohen’s *d* = 1.15) indicated that decision RTs were shorter in the instructed (919.08 ± 67.73 ms) vs. the free-choice condition (1410.35 ± 615.31 ms). Overall, this pattern of results suggests that choices that required longer decisions tended to be followed by longer interval estimations, thus lower implicit SoA. Subsequent correlational analysis, however, showed no relationship between decision RTs and *T*_scores_ (free condition: *r*(44) = .039, *p* = .799; instructed condition: *r*(44) = .043, *p* = .775).

## Discussion of Experiment

Overall, our results from experiment 1 replicate past findings by Kulakova et al. (2017), showing that the explicit and implicit measures of agency may dissociate. While explicit agency ratings were significantly higher in the free choice than the instructed condition and the win than the loss condition, implicit agency was not significantly different in the free choice compared with the instructed condition and in win compared with the loss condition.

The fact that the implicit measure of agency used here was not significantly different between conditions may seem counterintuitive. Possibly, the time estimation ratings are confounded by the shorter reaction times when choosing an option in the instructed condition, although we did not find any evidence for an association between decision RTs and *T*_scores_. The instructed trials are simpler and presumably require less effort, all of which may affect temporal judgements (Eagleman, 2008), and thus SoA. It is crucial to emphasize that agency is a multifaceted phenomenon that is co-constructed from a multitude of signals. These range from lower level efferent motor signals that relate to the execution of the action itself, to higher level action planning, and even to more abstract stages, such as the intention formation (Blakemore, Frith, & Wolpert, 1999; Moore, Wegner, & Haggard, 2009; Wegner, 2003). In this sense, one particular element of agentic behaviour that has been shown to influence the sense of agency and its measurement with intentional binding, namely the efference copy associated with the execution of a voluntary action (Haggard & Tsakiris, 2009), is present even in the instructed trials and, therefore, participants may retain a basic sense of agency.

Moreover, concerning the explicit agency ratings (VAS scores), we found an interaction effect with the cardiac cycle, indicating that when participants learned about the outcome of their choice during cardiac systole (i.e., enhanced cardiac signalling), they experienced lower sense of control of the outcomes than during cardiac diastole (i.e., lower cardiac signalling), but only when they made decisions freely (as opposed to being instructed).

Presentation at cardiac systole enhances perception of negative emotions, particularly rapidly presented fearful faces (Garfinkel et al., 2014), and to a lesser extent disgust (Gray et al., 2012). Exposure to threatening stimuli at cardiac systole compared to diastole, or when presented without reference to the cardiac cycle, facilitates extinction learning (Watson et al., 2019). Moreover, cardiac systole is also associated with enhanced expression of negative racial stereotypes (Azevedo et al., 2017). These results imply that cardiac systole is associated with enhanced perception of negative emotions; therefore, it might be associated more negatively than diastole. Possibly, this more ‘negative’ cardiac state, at systole, is associated with lower explicit control ratings when participants make the decisions freely, as opposed to being instructed what to do.

On the other hand, the timing of the outcome presentation did not affect implicit agency, suggesting that the state of cardiac signalling at the stage where participants learned about the outcome of their decision affected explicit but not implicit agency. Importantly, our initial finding with the cTET suggests that the time estimation per se, when no action is present, is not affected by the valence or timing of the sounds, excluding the possibility that the results regarding implicit agency are simply driven by the effects of cardiac timing on time perception.

## Experiment

In experiment 2, we aimed to investigate further the role of cardiac timing at the point of action making, on explicit and implicit measures of agency. For this purpose, we tracked when participants made their actions (i.e., pressed the key to indicate their choice) in each trial in relation to the cardiac cycle, and we assigned them as having been made within systole or diastole. During pilot testing (not reported here), we utilised a version of the task which had twice the number of trials (320). However, the length of the experiment led to high fatigue and increased inattention in participants. Due to these aspects as well as potential confounds associated with instructed trials as described above, only the free-choice condition was employed in experiment 2, with 160 trials. As a complementary analysis, we investigated the role of the heartbeat’s phase for self-initiated actions, to see whether agents implicitly act upon their environment so that relevant signals appear during preferred specific cardiac phases. Following past findings in the field (Kunzendorf et al., 2019), we expected to see an increased proportion of decision onsets being timed to occur during early phases of the cardiac cycle. For completeness, we also re-analysed the data from experiment 1 in the context of action-onsets. However, as this study was not initially designed to address this question and might, therefore, be underpowered, we report all the results in the Supplementary Materials.

## Methods

### Participants

Forty-seven participants (15 males; age 18–35, 23.60 ± 4.40 years old) completed the study. Participants were recruited from the Royal Holloway University community as well as from the Call for Participants (https://www.callforparticipants.com/) subject pool. Inclusion criteria comprised age between 18 and 35 years-old, normal or corrected-to-normal vision and hearing, and no current treatment for any mental or neurological disorders. Participants in our sample were not taking any medication (except for birth control), and we did not observe any abnormalities in their ECG signal. Participants gave written informed consent before taking part in the study and were reimbursed £10 per hour of their time and an additional £2 as winnings in the task. All procedures were approved by the local Ethics Committee and were conducted following The Code of Ethics of the World Medical Association.

### Task

The cardiac risky decision-making task was used, as described above. However, only the free-choice condition was used. Participants completed 160 trials, half of which were winning, half losing. On half the trials, the outcome was presented at systole and half at diastole (Fig. 2c). As in experiment 1, participants were free to make the choice whenever they felt like doing so. The timing of the key presses (hereafter referred to as the ‘action time’) was recorded on the ECG so that we could subsequently categorise whether the key press occurred during cardiac systole (between R peak and the end of T-wave) or cardiac diastole (between the end of T-wave and subsequent R peak) (see below).

### Data Analysis

A two-sided alpha level of 0.05 was used in all main statistical analyses. Bonferroni correction for multiple comparisons was applied for post hoc tests.

#### Behavioural Analysis

VAS ratings and *T*_scores_ were submitted to a 2 (outcome: win vs. lose) × 2 (outcome time: systole vs. diastole) × 2 (action time: systole vs. diastole) repeated measures ANOVA, performed in JASP (JASP Team, 2020).

#### Circular Analysis

We conducted an additional circular analysis to examine the relative onset of each key press (i.e., action time) throughout the entire cardiac cycle (from one R peak to the next) (Fig. 1). The analysis was conducted in R v3.6.0 (R Core Team, 2019) with R Studio v 1.1.442 (R Studio Team, 2016), according to procedures described previously (Kunzendorf et al., 2019) by adapting the R script accompanying that paper. Briefly, according to its relative timing within the RR interval, radian values between 0 and 2 π were assigned to each key-press time (action time). For each participant, we computed the circular mean of the circular distribution of actions. At the group level, we used a Rayleigh test for uniformity (Pewsey, Neuhäuser, & Ruxton, 2013) to test whether the spread of actions derived from a uniform distribution. For this purpose, we calculated mean vector via vector addition of individual means showing the average self-paced action time (key-press onset) in the cardiac cycle, across the group, and weighted this by its length (mean resultant length, *ϱ*) to reflect the spread of individual means around the cycle. The Rayleigh test measure ranges from 0 (indicating no) to 1 (indicating perfect) angular concentration. A statistically significant Rayleigh test indicates that the data are unlikely to be uniformly distributed around the circle (i.e., the cardiac cycle).

Since the Rayleigh test is a parametric method, we additionally used a non-parametric procedure to calculate confidence intervals and significance through bootstrapping (Ohl, Wohltat, Kliegl, Pollatos, & Engbert, 2016).

For each participant, we computed circular densities of actions (bandwidth = 20) and compared these circular densities with the null hypothesis of a uniform circular density. We drew a random bootstrap sample of 47 participants, with replacement. Confidence intervals (95%) were determined as 2.5% and 97.5% percentiles from the distribution of mean circular densities obtained by repeating the bootstrap procedure 10,000 times. Deviation from the circular uniform was considered significant when the 95% confidence interval determined by the bootstrapping was outside the circular density of a uniform distribution.

#### Binary Analysis

For the binary analysis, we segmented the cardiac cycle into systole (defined as the period between the R peak and the end of the T-wave; see Fig. 1) and diastole (defined as the period between the end of the T-wave and consecutive R peak). The segmentation was performed using an adapted script from the BioSigKit for ECG analysis (Sedghamiz, 2018) implemented in Matlab. The results were visualised and super-imposed on individual ECG traces to check for the accuracy of end of T-wave detection. Trials for which end of T-wave could not reliably be established were removed from the analysis (mean number of trials retained: 158.40 ± 3.17, range: 145–160). The self-paced key presses were then assigned to the respective cardiac phase (i.e., individual systole or diastole). To take into account the between-subject differences in heart rate (and thus cardiac phase lengths), we followed the procedures described previously (Kunzendorf et al., 2019). The sum of actions per phase (as a ratio of all valid trials per individual) was normalized to the proportion of the subject-specific phase length in the total cardiac cycle, using the formula: the normalised proportion of key presses for each cardiac phase = proportion of actions per cardiac phase/(individual cardiac phase length/individual mean R-R length).

If there is no cardiac effect, actions will be randomly distributed across both cardiac phases. That is, the rate of systolic (diastolic) actions should correspond to the proportion of systole (diastole) in the total R-R length, thereby resulting in a ratio of 1. A ratio > 1, and thus reflects an over-proportional accumulation of actions in the respective cardiac phase. In the group-level analysis, normalized systolic and diastolic ratios were tested against each other with a two-sided paired *t* test.

## Results

### Behavioural Results

#### Explicit Sense of Agency

**Fig. 4:**
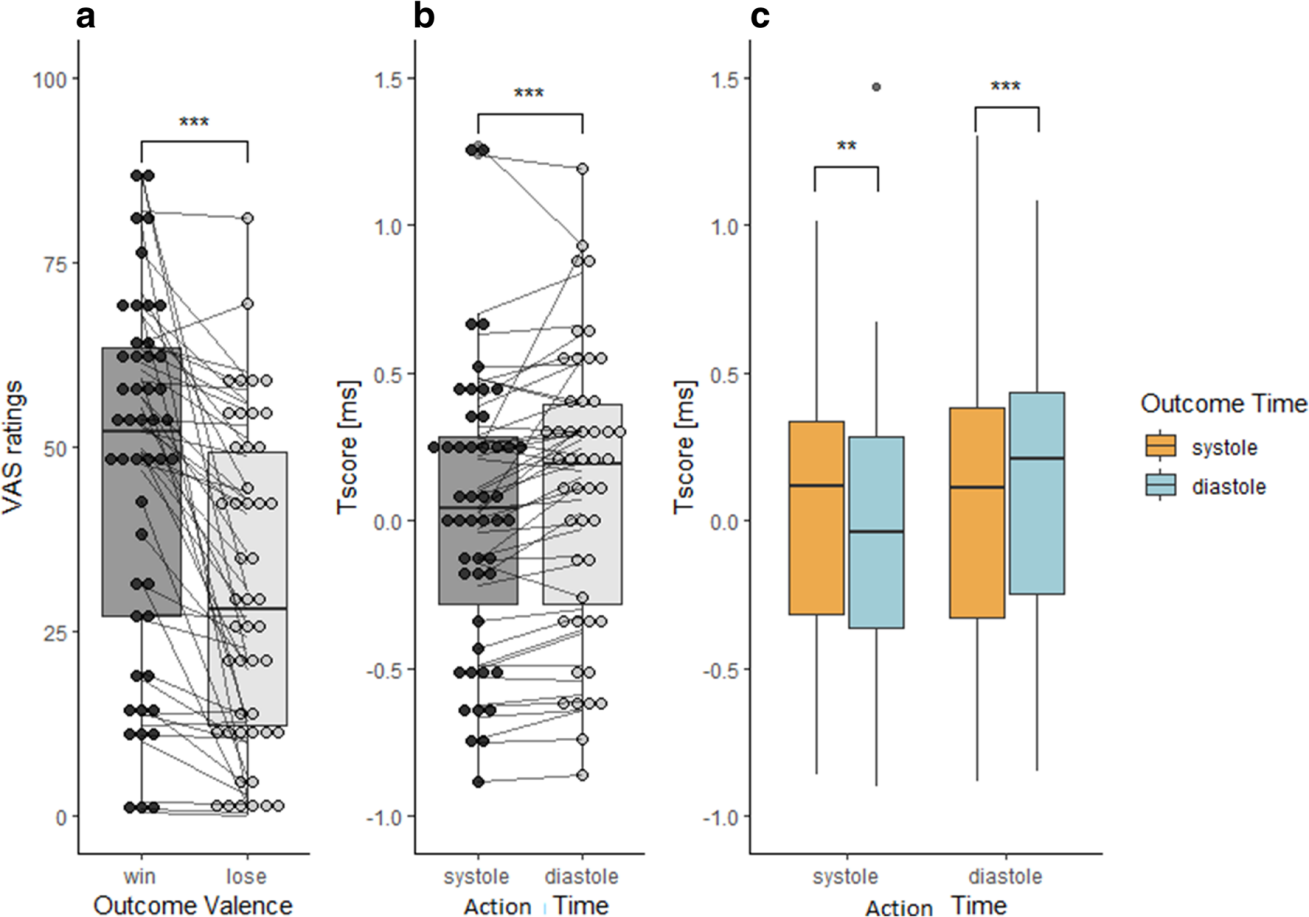
Results from experiment 2. The effect of outcome valence on VAS ratings (**a**). The effect of the action time (key-press timing) on *T*_scores_ (implicit agency measure) (**b**), as well as cardiac phase at action time × cardiac phase at outcome-time interaction (**c**)

Cronbach‘s *α* (Cronbach, 1951) for VAS control ratings equalled 0.97 with a 95% CI of (0.95, 0.98), again suggesting that the explicit agency measure has satisfactory internal consistency. We replicated the main effect of outcome valence on the VAS ratings (Fig. 4a), with higher agency ratings for the winning vs losing trials. No other main effects of interaction were found (see Table 3 for details).

**Table 3:** The results of the repeated measures ANOVA with VAS as dependent variable in experiment 2. Significant *p* values (< .0.05) are depicted in italics

	Sum of squares	df	Mean square	*F*	*p*	*η*^2^
Action time	0.00	1	0.00	0.00	.984	0.00
Residual	459.72	46	9.99			
Outcome time	8.47	1	8.47	0.96	.332	0.02
Residual	404.58	46	8.80			
Outcome	22,183.50	1	22,183.50	31.77	*< .001*	0.41
Residual	32,117.87	46	698.22			
Action time × outcome time	6.67	1	6.67	0.68	.413	0.02
Residual	448.68	46	9.75			
Action time × outcome valence	1.10	1	1.10	0.15	.697	0.00
Residual	329.60	46	7.17			
Outcome time × outcome valence	1.44	1	1.44	0.15	.699	0.00
Residual	437.48	46	9.51			
Action time × outcome time × outcome valence	0.02	1	0.02	0.00	.962	0.00
Residual	406.62	46	8.84			

Type III sum of squares

#### Implicit Sense of Agency

Cronbach‘s α (Cronbach, 1951) for *T*_scores_ equalled 0.98 with a 95% CI of (0.96, 0.99), again suggesting that the implicit agency measure has satisfactory internal consistency. There was a significant main effect of action time (key-press onset) on *T*_scores_ (Fig. 4b, Table 4), suggesting that the delay between action time and outcome delivery was judged as longer (i.e., agency was lower) when the action was made at diastole rather than systole. There was also a significant action-time × outcome-time interaction (Fig. 4c). Post hoc two-sided paired samples *t* tests were computed to follow-up the interaction. The Bonferroni correction for multiple comparisons was set to *p* < .013 (= .05/4). Post hoc tests revealed that, when the action was made within cardiac systole, the *T*_scores_ were higher (agency was lower) when the outcome was also presented at systole than diastole. When the action was made within cardiac diastole, the *T*_scores_ were higher (agency was lower) when the outcome was also presented at diastole than systole. Additionally, when the outcome was presented at diastole, *T*_scores_ were higher (agency was lower) when the action was made at diastole rather than systole. However, this comparison did not survive the correction for multiple comparisons (*p* = .021, see Table 5 for details).

**Table 4:** The results of the repeated measures ANOVA with *T*_scores_ as dependent variable for experiment 2. Significant *p* values (< .0.05) are depicted in italics

	Sum of squares	df	Mean square	*F*	*p*	*η*^2^
Action time	0.63	1	0.63	12.5	*< .001*	0.21
Residual	2.31	46	0.05			
Outcome time	0.02	1	0.02	0.42	.522	0.01
Residual	1.98	46	0.04			
Outcome	0.01	1	0.01	0.09	.766	0.00
Residual	3.00	46	0.07			
Action time × outcome time	0.79	1	0.79	10.77	*.002*	0.19
Residual	3.35	46	0.07			
Action time × outcome	0.00	1	0.00	0.01	.940	0.00
Residual	1.03	46	0.02			
Outcome time × outcome	0.01	1	0.01	0.44	.511	0.01
Residual	1.14	46	0.03			
Action time × outcome time × outcome valence	0.01	1	0.01	0.31	.578	0.01
Residual	1.24	46	0.03			

Type III sum of squares

**Table 5:** Post hoc tests on the action time x outcome time interaction on *T*_scores_. Significant comparisons after the Bonferroni correction for multiple comparisons (set at *p* < .013) are depicted in italics

*T*_scores_ comparison (action time–outcome time)			*t*	df	*p*	Mean Difference	SE Difference	95% CI		Cohen’s *d*
					(2-tailed, uncorrected)			Lower	Upper	
Systole-systole	-	systole-diastole	2.77	46	*.008*	0.11	0.04	0.03	0.18	0.40
Systole-systole	-	diastole-systole	0.33	46	.744	0.01	0.03	− 0.05	0.07	0.05
Diastole-diastole	-	diastole-systole	2.38	46	.021	0.08	0.03	0.01	0.14	0.35
Diastole-diastole	-	systole-diastole	4.05	46	*< .001*	0.17	0.04	0.09	0.26	0.59

#### Circular Analysis Results

**Fig. 5:**
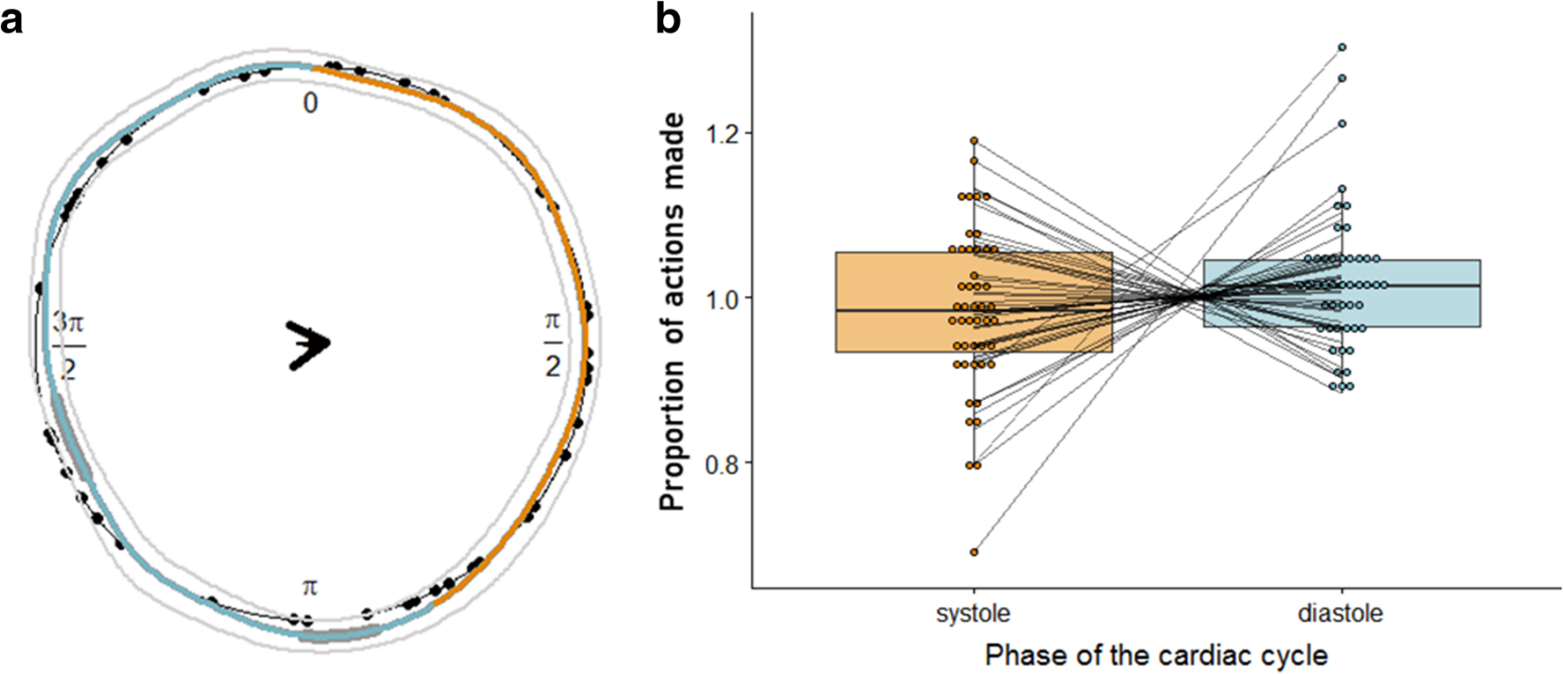
Circular (**a**) and binary (**b**) analysis of the timings of key presses (action timing) relative to the cardiac cycle. **a** Circular distribution of individual mean actions (black dots, *N* = 47) across the cardiac cycle (from R peak to R peak). On average, participants mainly made actions is the early phase of the cardiac cycle (weighted overall mean as black arrow). Based on a bootstrapping procedure, we computed the mean circular density of picture onsets (coloured line) as well as a 95% CI (within inner and outer thin grey lines). Segments of the cardiac cycle are determined as statistically significant (thick grey segments) when the circular density significantly differs from the circular uniform (i.e., the lower bound of the CI is outside of the black uniform circle). To relate segments of the cardiac cycle to the two cardiac phases (systole = orange, diastole = blue), overall mean systole and diastole lengths were obtained, showing that both of the significant density segments fall into diastole. **b** The relative proportion of actions for systole and diastole. The proportions of actions were normalised for the duration of systole and diastole, respectively

The distribution of self-paced actions relative to the cardiac R-R interval showed, overall, that the average responses fell around the first quarter of the cardiac cycle (*M* = 0.47 *π*, SE = 0.22 *π*, *ϱ* = 0.02 ± 0.77 *π*; Fig. 5a). The inferential circular statistics indicated that in the present experiment the key-presses timings are likely to be uniformly distributed (Rayleigh’s test of uniformity, *R*_0_ = 0.05, *p* = .869). Non-parametric bootstrapping, on the other hand, showed that participants made significantly more actions in a narrow period that was half-way through the cardiac cycle (at the beginning of the diastole, between 0.93 and 1.00 π) and made significantly fewer actions in the later stage of the cycle (mid-diastole, between 1.35 and 1.43 π; Fig. 5a). Noteworthy, both of these significant density segments fell within cardiac diastole. Importantly, we also observed homogenous distribution of action times across the cardiac cycle in experiment 1 (see Supplementary Materials for details).

#### Binary Analysis Results

The binary analysis showed no significant differences between the (normalised) proportion of responses made during systole and diastole (Fig. 5b; *t*(46) = 1.36, *p* = .182, *d* = 0.20; systole: *M* = 0.98, SD = 0.01; diastole: *M* = 1.02, SD = 0.09). Therefore, we conclude that participants did not show a preference for making actions at either systole or diastole.

## Discussion of Experiment

Our results suggest that the timing of action relative to the cardiac cycle affects implicit agency measures. Implicit agency was higher when the actions were made within cardiac systole (a state of enhanced interoceptive signalling) than at diastole. Moreover, we observed an interaction between the action timing and timing of the presentation of the outcome, indicating that congruency in the timing of action and its effect (i.e., systole-systole or diastole-diastole) was associated with lower intentional binding (lower agency) than incongruent timing (i.e., systole-diastole or diastole-systole). These findings suggest that both the heightened, as well as the diminished, state of cardiac signalling associated with both action time and outcome delivery time leads to lower binding, therefore, lower sense of agency. Importantly, we did not find any evidence of cardiac impact on explicit agency ratings, suggesting distinct effects of interoceptive signals on explicit and implicit agency.

Furthermore, the analysis of action timings revealed that the action timings were equally distributed around the cardiac cycle. There were no differences in the proportion of actions made at the two cardiac phases (systole and diastole).

## General Discussion

Across two experiments, we studied the effects on implicit and explicit SoA measures of several factors, namely: choice type; outcome valence; cardiac arousal at the stage of action time; and learning about the outcome of one’s choice. Additionally, we investigated the timings of freely-initiated actions in relation to the cardiac cycle, to see whether participants made actions preferentially in any particular cardiac phases. Across these two experiments, we were able to replicate several findings: Firstly, (1) we found in both experiments an effect of cardiac phase at the time of action-making on implicit SoA measure (T_scores_), indicating that the SoA is higher when the action is made within cardiac systole than diastole. Secondly, (2) regarding the implicit SoA measure, a congruent cardiac state signalling (conveying information about the state of cardiovascular arousal) during action and outcome phase (i.e., systole and systole or diastole and diastole) was associated with lower SoA levels, while an incongruent cardiac state signalling (i.e., systole and diastole or diastole and systole) was associated with enhanced agency. Thirdly, (3) across both experiments, implicit SoA was not affected by the outcome valence (win vs loss). Furthermore, (4) we observed enhanced explicit SoA judgements (VAS control ratings) following wins relative to losses. Moreover, (5) there was no such effect of cardiac phase on explicit SoA. Finally, (6) the distribution of freely-initiated actions was uniform across the cardiac cycle.

It seems that interoceptive signalling at the stage of making an action has a pronounced impact on implicit agency (point 1 above). Intentional binding was higher when the action (key press) was made within cardiac systole rather than diastole, suggesting that higher cardiac arousal signalling during action time is related to higher implicit sense of agency. This is in agreement with previous results showing that unspecific arousal states (induced by colours of the stimuli or physical effort to exert an action) related to the stage of the action itself increases people’s implicit SoA over their actions (Minohara et al., 2016; Wen, Yamashita, & Asama, 2015). In contrast, the action timing in relation to the cardiac cycle did not affect explicit agency ratings in either of the studies (point 5 above).

Moreover, our results indicate an interaction between the cardiac arousal state during the action time and the outcome-learning time (point 2 above). In trials when the action was made during systole, agency was higher when the outcome was revealed at diastole compared to systole, and vice versa. These findings suggest that a congruent high or low interoceptive signalling during action time and outcome-time results in lower implicit agency.

In the context of learning, the optimal ‘mid’ state of arousal is often taken to imply that inverted U-shaped relationships exist generally between physiological arousal and cognitive performance (Hebb, 1955; Yerkes & Dodson, 1908). Therefore, excessively high or low arousal might lead to suboptimal behavioural/cognitive performance and interoceptive cues of current cardiovascular arousal could also similarly affect SoA. In support of this, a recent study showed that arousal states associated with negative emotional valence, such as fear of receiving an electric shock or anger induced by a frustrating task, reduce the implicit sense of control over an action outcome relative to neutral state, even though the objective causal link between action and outcome remains the same (Christensen, Di Costa, Beck, & Haggard, 2019). Thus, generalized elevated negative emotional arousal (during both action and outcome-learning phase) decreases implicit agency. Similarly, physical or mental strain decreases temporal binding, relative to a control condition (Howard, Edwards, & Bayliss, 2016).

While enhanced explicit agency judgements following wins vs losses (point 4 above) is a consistently reported finding (e.g., Barlas et al., 2018; Kulakova et al., 2017), past evidence regarding the effect of outcome valence (point 3 above) on intentional binding is mixed. For example, when faced with unpredictable outcomes, participants show higher intentional binding (stronger sense of agency) for negative outcomes determined by their free choice. Conversely, when outcome valence was predictable, such interaction was not observed (Tanaka & Kawabata, 2019). Others reported higher intentional binding on the trials in which actions were followed by negative (no rewards) vs positive (reward) outcomes (Di Costa, Théro, Chambon, & Haggard, 2018). Possibly, the stronger sense of agency on negative outcomes determined by their free choice (unlike a fixed option) play a role in learning adaptive behaviours (Tanaka & Kawabata, 2019). Contrary findings by Yoshie and Haggard (2013) suggest that negative emotional outcomes attenuate SoA over voluntary actions relative to neutral or positive outcomes. However, these results were not replicated by a different group (Moreton et al., 2017), but rather indicated that the outcome valence had little influence on intentional binding. Importantly, in agreement with Moreton et al. (2017) we found no significant differences in implicit agency measures following wins and losses, suggesting that while explicit agency seems to diminish following negative outcomes, outcome valence has little effect on implicit agency measures. Together, our findings indicate that self-reported agency and temporal biding reflect different aspects of the sense of agency (Dewey & Knoblich, 2014; Saito, Takahata, Murai, & Takahashi, 2015; cf. Imaizumi & Tanno, 2019).

Finally, we also probed the association between the cardiac cycle and the self-paced action-making (key presses; point 6 above). Given a growing body of evidence showing that behavioural, cognitive and somatosensory processing differs depending on the phase of the cardiac cycle (Galvez-Pol, McConnell, & Kilner, 2020; Garfinkel et al., 2014; Kunzendorf et al., 2019; Motyka et al., 2019; Ohl et al., 2016; Rae et al., 2018), one could expect similar biases for the action timings with relation to the cardiac cycle. In contrast, in both of our experiments, the key presses (action times) were fairly equally distributed around the cardiac cycle. It has been suggested that an internal central pacemaker guides the timing of motor actions (Coleman, 1921; McCraty, Atkinson, Tomasino, & Bradley, 2009; Treisman, Faulkner, Naish, & Brogan, 1990). However, the benefits of timing our behaviours to the cardiac cycle remain speculative. Possibly, agents act in a way to maximize the likelihood of relevant signals being processed during optimal phases of the cardiac cycle (Kunzendorf et al., 2019). Yet, our data from the circular analysis suggests that such an optimal phase of the cardiac cycle might be not present for action-making onsets. Possibly, initiating actions may be more related to other types of interoceptive signalling, for example breathing phase, with recent evidence suggesting that voluntary actions are more often initiated during exhalation than inhalation and do not differ across cardiac phases (Park et al., 2020).

Notably, one finding did not replicate across the two experiments. In Experiment 1, we found a choice type by outcome-time interaction, suggesting that when decisions were made freely, but not instructed, explicit agency ratings were higher if the outcome was presented during cardiac diastole than systole. In Experiment 2, with only free-choice decisions present, such effect was not present. Noteworthy, the agentic context of Experiment 1 was quite different from that of Experiment 2. In Experiment 1 there were trials where agency was accentuated, as participants were free to choose, and ‘instructed’ trials involving a diminished agency. Agency literature has shown important contextual effects on agency, such as: prior intentions (Moore et al., 2009); congruency between sensory-motor signals (Blakemore et al., 1999) and degrees of discrepancy; and predictive versus postdictive processes (Wegner, 2003). It may, therefore, be the case that the observed cardiac effects on the free trials arise when contrasted with the instructed trials, where (by definition) the levels of experienced agency would fluctuate substantially across the experiment ─ from a higher sense of explicit agency in free trials and lower levels of explicit agency in instructed trials. Future studies should address this issue.

Noteworthy, Experiment 2 is not a direct replication of Experiment 1; instead, it answers a complementary question. Therefore, the scope for direct comparison of the findings across the two experiments is limited. Nevertheless, and with this limitation in mind, we do replicate the findings of the action timing and action timing by outcome timing interaction in implicit agency ratings across both experiments (see Supplementary Materials for details).

Importantly, the implicit and explicit measures of agency poorly correlated with each other in the current study (see Supplementary Materials for details). Based on past findings, it remains unclear whether implicit and explicit agency measures reflect the same or different constructs of agency. Some research indicates that both measures coincide, while others report no correlation between the two (Dewey & Knoblich, 2014; Imaizumi & Tanno, 2019; Saito et al., 2015). Our results suggest that implicit and explicit measures of agency might reflect distinct constructs of agency. Moreover, although the intentional binding effect is widely used in agency research (see Moore & Obhi, 2012, for a review), the use of this method as an implicit measure of agency has recently been criticized as merely reflecting the multisensory causal binding, without the necessity of intentional action (Suzuki, Lush, Seth, & Roseboom, 2019). In this light, our findings of the role of cardiac timing on temporal binding might be interesting for the field of multisensory integration research.

Some limitations merit comment. We did not screen participants for underlying heart/autonomic function conditions, which may have possibly affected the results. However, participants were young, not taking any medication (except for birth control), and we did not observe any abnormalities in the ECG signal. Finally, by tapping into the extremes of ongoing fluctuations of baroreceptor activity which co-vary with the cardiac cycle and in line with previous studies employing similar methodologies (e.g., Azevedo et al., 2017; Garfinkel et al., 2014; Gray et al., 2012), we interpreted our findings in context of the central representation of cardiovascular arousal. However, with each heartbeat, there is likely exteroceptive sensory information being conveyed from muscle and skin receptors. In this way, skin and muscle receptors signalling could also be contributing to any behavioural outcomes, in parallel to what is conveyed via the baroreceptors. Therefore, the effects reported here may not only be explained through purely interoceptive influences but also through somatosensory mechanisms. Indeed, the experience of the cardiovascular state of the body is likely mediated via both interoceptive and somatosensory afferent pathways (Khalsa, Rudrauf, Feinstein, & Tranel, 2009).

In conclusion, our results provide evidence for the influence of interoceptive processing on the sense of agency, adding to a growing field of literature on interoception and volition (Galvez-Pol et al., 2020; Kunzendorf et al., 2019; Ohl et al., 2016). In particular, we show that explicit (ratings of control) and implicit (temporal judgement) measures of agency are differentially affected by factors such as choice type (free vs instructed); outcome valence; and cardiac arousal. Thus, implicit and explicit agency may reflect distinct aspects of agency or causality judgements. Overall, our results provide experimental support for a recent theoretical model proposing that the SoA depends on the integration of internal bodily cues with exteroceptive sensory signals (Seth et al., 2012; Seth & Tsakiris, 2018) and offering changes in arterial baroreceptors activity as a mechanistic account for the model. Future research should investigate the neural underpinnings of these effects in decision-making.

## Electronic supplementary material

ESM 1 (DOCX 173 kb)

## Additional Information

### Data Availability

The data produced by the research is available here: https://osf.io/3swjm/?view_only=21c835b55bbd4eae92ab51d0263b42fb
