# Supplementary material for: Feeling in Control: The Role of Cardiac Timing in the Sense of Agency
Source: Affect Sci. 2020 Aug 28;1(3):155–71. doi: 10.1007/s42761-020-00013-x (PMC9382947; doi:10.1007/s42761-020-00013-x)
Supplement: Supplementary file 1 — (DOCX 173 kb) [file 42761_2020_13_MOESM1_ESM.docx]

Feeling in control: The role of cardiac timing in the sense of agency

Aleksandra M. Herman^1^* & Manos Tsakiris^1,2^

^1^Lab of Action and Body, School of Psychology, Royal Holloway University of London

^2^The Warburg Institute, University of London

* Corresponding Author

Email: aleksandra.herman@sussex.ac.uk

Address: Royal Holloway, University of London, Egham, Surrey, TW20 0EX

# Supplementary Materials

Below we report the analyses regarding the effect of cardiac timing during action-time on the sense of agency (SoA) in Experiment 1. For the purposes of these analyses, we traced when participants made their actions (i.e. pressed a key to indicate their decision regarding which stimuli to choose) in relation to the cardiac cycle following the procedures described in the manuscript. Three participants were excluded from the analyses completely as reliable identification of the cardiac phases was not possible on the substantial proportion of trials (>15% trials per participant excluded). Therefore, the sample for analyses reported here is 43 individuals (35 females).

Additionally, we also report inter-subject correlation coefficients (Spearman’s r, two-tailed) between the implicit (Tscores) and explicit (VAS ratings) agency measures for Experiment 1 and 2.

# 2x2x2x2 RM ANOVA

To investigate the role of cardiac phase during action-time on the SoA in Experiment 1, we conducted 2 (action time: systole vs diastole) x 2 (outcome time: systole vs diastole) x 2 (choice type: free vs instructed) x 2 (outcome valence; win vs lose) repeated measures ANOVA with VAS score (explicit agency) and Tscores (implicit agency) as dependent variables.

## Explicit Agency Ratings

Regarding explicit agency ratings, repeated-measures ANOVA results were unchanged from the analysis reported in the manuscript: there was the main effect of choice (free > instructed) and outcome type (win > lose), see Supplementary Table 1 (STable 1) for details. The choice x outcome time interaction was also significant (free choice with outcome at diastole > free choice with outcome at systole). There were no significant results regarding the action time on explicit agency ratings.

STable 1 The results of the 2x2x2x2 repeated measures ANOVA comparing explicit agency ratings (VAS scores).

|  | **Sum of Squares** | **df** | **Mean Square** | ***F*** | ***P***  ***(2-tailed)*** | **η_p_^2^** |
| --- | --- | --- | --- | --- | --- | --- |
| action time | 33.60 | 1 | 33.60 | 0.67 | .417 | 0.02 |
| Residual | 2098.45 | 42 | 49.96 |  |  |  |
| **outcome valence** | **12138.77** | **1** | **12138.77** | **32.00** | **< .001** | **0.43** |
| Residual | 15932.43 | 42 | 379.34 |  |  |  |
| **choice** | **57731.66** | **1** | **57731.66** | **34.34** | **< .001** | **0.45** |
| Residual | 70603.19 | 42 | 1681.03 |  |  |  |
| outcome time | 26.52 | 1 | 26.52 | 0.63 | .433 | 0.02 |
| Residual | 1777.52 | 42 | 42.32 |  |  |  |
| action time * outcome valence | 1.03 | 1 | 1.03 | 0.04 | .850 | 0.00 |
| Residual | 1193.65 | 42 | 28.42 |  |  |  |
| action time * choice | 45.73 | 1 | 45.73 | 1.63 | .209 | 0.04 |
| Residual | 1177.49 | 42 | 28.04 |  |  |  |
| outcome valence * choice | 8.80 | 1 | 8.80 | 0.13 | .726 | 0.00 |
| Residual | 2965.13 | 42 | 70.60 |  |  |  |
| action time * outcome valence * choice | 35.30 | 1 | 35.30 | 0.79 | .380 | 0.02 |
| Residual | 1880.39 | 42 | 44.77 |  |  |  |
| action time * outcome time | 6.67 | 1 | 6.67 | 0.21 | .652 | 0.01 |
| Residual | 1361.61 | 42 | 32.42 |  |  |  |
| outcome valence * outcome time | 18.91 | 1 | 18.91 | 0.80 | .375 | 0.02 |
| Residual | 987.60 | 42 | 23.51 |  |  |  |
| action time * outcome valence * outcome time | 72.26 | 1 | 72.26 | 3.31 | .076 | 0.07 |
| Residual | 916.86 | 42 | 21.83 |  |  |  |
| **choice * outcome time** | **207.23** | **1** | **207.23** | **4.37** | **.043** | **0.09** |
| Residual | 1991.96 | 42 | 47.43 |  |  |  |
| action time * choice * outcome time | 59.48 | 1 | 59.48 | 1.36 | .250 | 0.03 |
| Residual | 1834.51 | 42 | 43.68 |  |  |  |
| outcome valence * choice * outcome time | 14.50 | 1 | 14.50 | 0.29 | .591 | 0.01 |
| Residual | 2071.05 | 42 | 49.31 |  |  |  |
| action time * outcome valence * choice * outcome time | 21.01 | 1 | 21.01 | 0.65 | .426 | 0.02 |
| Residual | 1365.11 | 42 | 32.50 |  |  |  |

Note. Type III Sum of Squares

## Implicit Agency Ratings

The findings regarding implicit agency measure are reported in STable 2. We found the main effect of action time, indicating that the Tscores were lower (i.e., the agency was higher) when the action was made during cardiac systole (-.0464±0.42) than diastole (-.0195±0.43) (SFigure 1A). There was also a significant effect of choice type, indicating that the Tscores were lower (the agency was higher) during the instructed (-.0508±0.42) than free (-.0151±0.43) trials (SFigure 1B). There was also an action time x outcome time interaction. We employed paired-samples *t*-tests to follow-up on this interaction effect. With four planned comparisons, significance value corrected for multiple comparisons (Bonferroni) was set to *p* < .013. The outcomes are summarised in SFigure 1C and STable 3. The results suggest that if participants made their actions during cardiac systole, the Tscores were lower (therefore, the agency was higher) if the outcomes were presented during cardiac diastole than during systole. In contrast, if participants made their actions during cardiac diastole, the Tscores were lower (therefore, the agency was higher) if the outcomes were presented during cardiac systole than during diastole. Moreover, when the outcome was presented on cardiac systole, the Tscores were lower when the actions were made during diastole than systole. The reverse pattern was seen for the outcomes presented at diastole. These results suggest that congruent state of cardiovascular arousal during both action phase and outcome delivery phase is associated with diminished SoA, while incongruent state of cardiovascular arousal is related to elevated SoA.

STable 2 The results of the 2x2x2x2 repeated measures ANOVA comparing implicit agency (Tscores).

|  | **Sum of Squares** | **df** | **Mean Square** | ***F*** | ***P***  ***(2-tailed)*** | **η_p_^2^** |
| --- | --- | --- | --- | --- | --- | --- |
| **action time** | **0.12** | **1** | **0.12** | **4.55** | **.039** | **0.10** |
| Residual | 1.15 | 42 | 0.03 |  |  |  |
| outcome valence | 0.52 | 1 | 0.52 | 2.58 | .116 | 0.06 |
| Residual | 8.49 | 42 | 0.20 |  |  |  |
| **choice** | **0.22** | **1** | **0.22** | **4.25** | **.045** | **0.09** |
| Residual | 2.16 | 42 | 0.05 |  |  |  |
| outcome time | 0.07 | 1 | 0.07 | 2.64 | .112 | 0.06 |
| Residual | 1.17 | 42 | 0.03 |  |  |  |
| action time * outcome valence | 0.04 | 1 | 0.04 | 1.86 | .180 | 0.04 |
| Residual | 0.91 | 42 | 0.02 |  |  |  |
| action time * choice | 0.01 | 1 | 0.01 | 0.23 | .636 | 0.01 |
| Residual | 0.84 | 42 | 0.02 |  |  |  |
| outcome valence * choice | 0.01 | 1 | 0.01 | 0.80 | .375 | 0.02 |
| Residual | 0.75 | 42 | 0.02 |  |  |  |
| action time * outcome valence * choice | 0.00 | 1 | 0.00 | 0.14 | .706 | 0.00 |
| Residual | 1.26 | 42 | 0.03 |  |  |  |
| **action time * outcome time** | **2.36** | **1** | **2.36** | **22.84** | **< .001** | **0.35** |
| Residual | 4.34 | 42 | 0.10 |  |  |  |
| outcome valence * outcome time | 0.02 | 1 | 0.02 | 0.59 | .448 | 0.01 |
| Residual | 1.08 | 42 | 0.03 |  |  |  |
| action time * outcome valence * outcome time | 0.01 | 1 | 0.01 | 0.26 | .612 | 0.01 |
| Residual | 1.39 | 42 | 0.03 |  |  |  |
| choice * outcome time | 0.09 | 1 | 0.09 | 3.35 | .074 | 0.07 |
| Residual | 1.16 | 42 | 0.03 |  |  |  |
| action time * choice * outcome time | 0.02 | 1 | 0.02 | 0.92 | .343 | 0.02 |
| Residual | 0.83 | 42 | 0.02 |  |  |  |
| outcome valence * choice * outcome time | 0.02 | 1 | 0.02 | 1.30 | .260 | 0.03 |
| Residual | 0.53 | 42 | 0.01 |  |  |  |
| action time * outcome valence * choice * outcome time | 0.02 | 1 | 0.02 | 1.28 | .264 | 0.03 |
| Residual | 0.71 | 42 | 0.02 |  |  |  |
| *Note.*  Type III Sum of Squares |  |  |  |  |  |  |


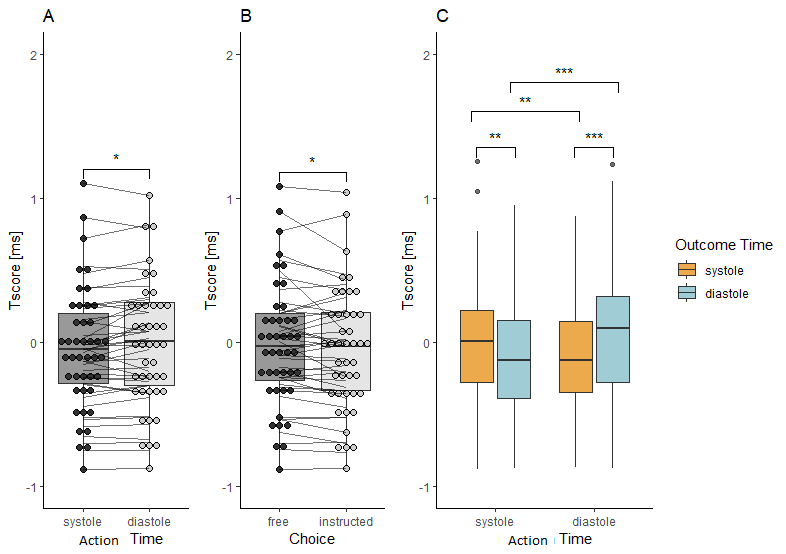


SFigure 1 The effect of action time on Tscores (A), the effect of choice type of Tscores (B), as well as cardiac phase at action-time vs outcome time interaction (C) on Tscores (implicit agency).

STable 3 Post-hoc tests on the action time x outcome time interaction on Tscores. Significant comparisons after the Bonferroni correction for multiple comparisons (set at p < .013) are depicted in bold.

| **Tscores comparison** | | | ***t*** | **df** | ***p*** | **Paired Differences** | | **95% CI** | |
| --- | --- | --- | --- | --- | --- | --- | --- | --- | --- |
| **(action time -outcome time)** | | |  |  | **(2-tailed, uncorrected)** | **Mean** | **SEM** | **Lower** | **Upper** |
| systole-systole | vs | systole-diastole | 3.58 | 43 | **.001** | 0.10 | 0.03 | 0.04 | 0.15 |
| diastole-systole | vs | diastole-diastole | -5.12 | 43 | **< .001** | -0.14 | 0.03 | -0.19 | -0.08 |
| systole-systole | vs | diastole-systole | 3.53 | 43 | **.001** | 0.09 | 0.03 | 0.04 | 0.14 |
| systole-diastole | vs | diastole-diastole | -5.11 | 43 | **< .001** | -0.14 | 0.03 | -0.20 | -0.09 |

# Action-time onsets analysis

As a complementary analysis, we investigated whether there were any biases in key presses (i.e., action times) in relation to the cardiac cycle following procedures described in details in the manuscript.

## Circular analysis

The distribution of self-paced actions relative to the cardiac RR interval showed overall that on average participants responded most often in the second quarter of the cardiac cycle (M = 0.64 π, SE = 0.21 π, ϱ = 0.03 ± 0.72; SFigure 2A). Inferential circular statistics indicated that in the present experiment the action onsets (key-presses timings) are likely to be uniformly distributed (Rayleigh test statistics, R0 = 0.08, *p* = .748) and this conclusion was also supported by the nonparametric bootstrapping analysis (SFigure 2A).

Noteworthy, analysing actions timing for free and instructed choices separately, confirmed the uniformly distributed pattern of actions for both free (R0 = 0.20, *p* = .170; M = 0.74 π, SE = 0.17 π, ϱ = 0.06 ± 0.57; SFigure 2B) and instructed choices (R0 = 0.07, *p* = .822; M = -0.59 π, SE = 0.22 π, ϱ = 0.02 ± 0.74; SFigure 2C).


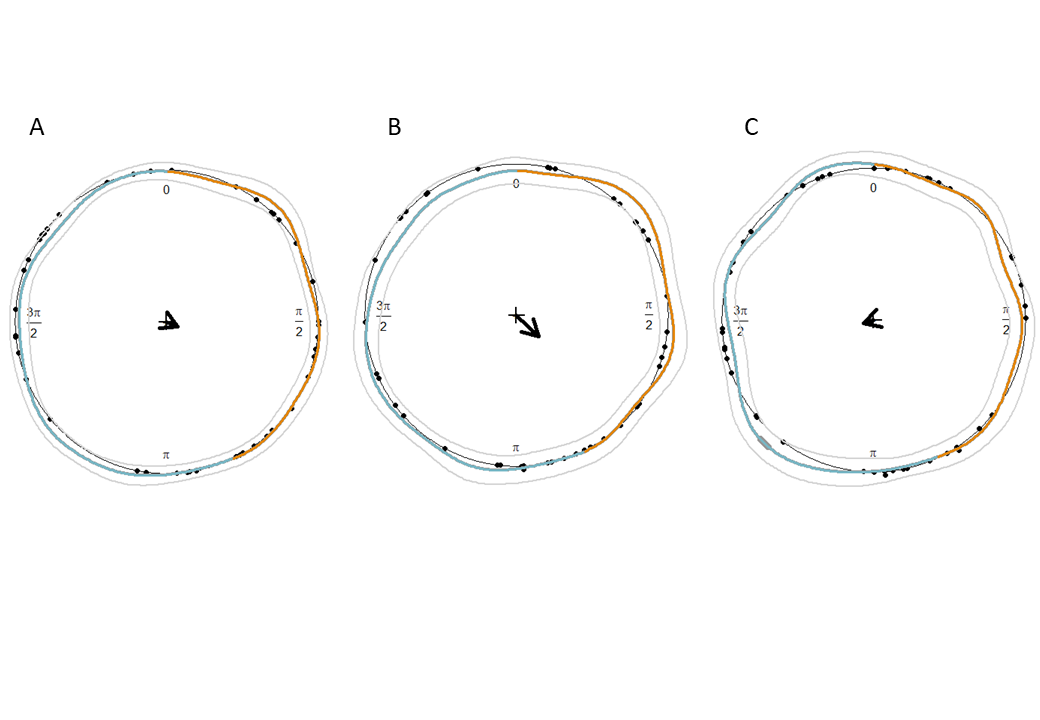


SFigure 2 Circular analysis of actions-making relative to the cardiac cycle. Circular distribution of individual mean actions (black dots, N = 43) across the cardiac cycle (from R peak to R peak) for A) all trials considered together, B) free choice only, C) instructed choice only. We observed on average more self‐prompted actions (weighted overall mean as black arrow) in the middle of the cardiac cycle. Based on a bootstrapping procedure, we computed the mean circular density of actions onsets (thicker lines) as well as a 95% CI (within inner and outer thin grey lines). Segments of the cardiac cycle are determined as statistically significant (thick grey segments) when the circular density significantly differs from the circular uniform (i.e., the lower bound of the CI is outside of the black uniform circle). To relate segments of the cardiac cycle to the two cardiac phases, colours are used (systole = orange, diastole = blue).

# Correlations between implicit and explicit measures of agency

For completeness, we also computed inter-subject correlations between implicit and explicit agency measures for each condition (Spearman’s rho, two-tailed). As high VAS ratings indicate high SoA, while high Tscores indicate poor temporal binding (i.e. low SoA), if the two indexes tap onto the same construct, we would expect a high negative correlation between them. Overall, implicit and explicit measures correlated rather poorly with no correlation reaching statistical significance (STable 4). The correlation coefficients range from very weak to weak according to Evans' (1996) guidelines. These results suggest that implicit and explicit measures of agency might reflect distinct constructs of agency.

STable 4 Inter-subject correlation coefficients between implicit (Tscores) and explicit (VAS ratings) agency measures for each condition of Experiment 1 and 2. Note: the p-values are uncorrected for multiple comparisons.

| **Experiment 1** | | |  | **Experiment 2** | | |
| --- | --- | --- | --- | --- | --- | --- |
| **Condition (choice type, outcome time, outcome valence)** | **Spearman's rho** | **p-value** |  | **Condition (action time, outcome time, outcome valence)** | **Spearman's rho** | **p-value** |
| instructed, diastole, win | .011 | .942 |  | systole, diastole, win | -.279 | .057 |
| instructed, systole, win | -.023 | .646 |  | systole, systole, win | -.263 | .075 |
| free, diastole, win | .070 | .881 |  | systole, diastole, lose | -.262 | .075 |
| instructed, diastole, lose | -.165 | .273 |  | diastole, diastole, win | -.226 | .127 |
| instructed, systole, lose | -.129 | .393 |  | diastole, systole, win | -.217 | .142 |
| free, systole, win | .087 | .562 |  | systole, systole, lose | -.090 | .549 |
| free, diastole, lose | .061 | .688 |  | diastole, diastole, lose | -.062 | .681 |
| free, systole, lose | .021 | .891 |  | diastole, systole, lose | -.014 | .925 |
|  |  |  |  |  |  |  |

# Supplementary References:

Evans, J. D. (1996). *Straightforward Statistics for the Behavioral Sciences*. *Pearson’s correlation*. Pacific Grove, CA: Brooks/Cole Publishing.
